# Coexistent hairy cell leukaemia and hepatosplenic t-cell lymphoma: a case report

**DOI:** 10.1186/1746-1596-9-58

**Published:** 2014-03-12

**Authors:** Gorana Gasljevic, Veronika Kloboves-Prevodnik, Barbara Gazic, Marjeta Vovk

**Affiliations:** 1 Department of Pathology, Institute of Oncology, Zaloska 2, Ljubljana 1000, Slovenia; 2 Department of Cytology, Institute of Oncology, Zaloska 2, Ljubljana 1000, Slovenia; 3 Department of internal medicine, Institute of Oncology, Zaloska 2, Ljubljana 1000, Slovenia

**Keywords:** Hairy cell leukemia, Hepatosplenic γɗ T cell lymphoma, Coexistency, Flow cytometric immunophenotyping

## Abstract

**Background:**

Hairy cell leukaemia (HCL) is a chronic B-cell leukaemia characterized by expansion of neoplastic cells in the spleen, bone marrow and blood. Symptoms of HCL are related to pancytopenia and immune deficiency. Patients with HCL have an increased risk of second malignancy either in a form of synchronous disease or in a form of an increased incidence of a second neoplasm after the treatment of HCL. Hepatosplenic T-cell lymphoma (HSTCL) is a rare form of aggressive extranodal T-cell lymphoma. Its pathogenesis is connected to a chronic immune deficiency status and its coexistence with other neoplasms is practically non-existent.

**Case:**

We present a case of a 53-year-old female patient suffering from hepatosplenomegaly, peripheral lymphadenopathy and related B symptoms. An excisional biopsy of the enlarged axillary lymph node revealed partial infiltration with CD3+/CD56+/TIA + T cell lymphoma. Bone marrow trephine biopsy and flow cytometric immunophenotypization of bone marrow cells and peripheral blood showed presence of two types of neoplastic cells in the peripheral blood and in the bone marrow (composite lymphoma). One of them showed typical morphologic characteristics and immunohistochemical features of HCL, while another one was morphologically and immunophenotypically consistent with the diagnosis of HSTCL, respectively. The patient was treated with multivalent chemotherapy including rituximab but all treatments turned out to be only partially effective. While HCL responded to the treatment, HSTCL was refractory to the chemotherapy and the patient died 7 months after the initial diagnosis because of haematemesis induced by Mallory-Weiss syndrome.

**Conclusion:**

This is the first recorded case of coexistent HCL and HSTCL in the same patient. A multidisciplinary approach, encompassing careful morphology interpretation, immunophenotypic, cytogenetic and molecular analyses, is mandatory to obtain an accurate diagnosis of composite lymphoma.

**Virtual slides:**

The virtual slides for this article can be found here: http://www.diagnosticpathology.diagnomx.eu/vs/9354870531161685.

## Background

Hairy cell leukaemia (HCL) is a chronic B-cell leukaemia characterised by pancytopenia and clonal expansion of mature malignant B cells in the spleen, bone marrow and blood [1]. Characteristically, patients with HCL do not suffer from leukaemia itself but from symptoms that are related to pancytopenia and opportunistic infections [2]. Hepatosplenic T-cell lymphoma (HSTCL) is a rare form of extranodal T-cell lymphoma derived from cytotoxic T cells. In up to 20% of cases, its pathogenesis is connected to a chronic immune deficiency status, such as prolonged immunosuppressive therapy after organ transplantation, long-term combined therapy with thiopurines and anti-TNF agents for chronic inflammatory bowel diseases or prolonged antigen stimulation [1,3]. Both diseases demonstrate propensity to infiltrate the spleen, liver and bone marrow. Their coexistence has to be exceptional because we have found no reports about concurrent occurrence of HCL and HSTCL in the English literature by now. The objective of this study is to report the clinical and pathological features of a 53-year-old female patient with coexistent HCL and HSTCL who was recently treated at our institution.

## Case report

A 53-year-old female was referred to our centre because of a two-month lasting exhaustion, abdominal distension, early satiety and fever of up to 39°C. Her medical history was not significant. She had been travelling for a longer periods of time in the countries of the Middle and Far East, especially to Indochina and India. Physical examination showed splenomegaly and hepatomegaly, palpable at 15 and 6 cm below the costal margin, respectively. Peripheral lymph nodes measured up to 1 cm in the largest diameter. Petechiae and suffusions were present on the skin of the thoracic region and flanks. Laboratory tests demonstrated: (1) white blood count 3.40 × 10^9^/l (Neu 2,01 (59%) , Ly 0,99 (29%), Mono 0,17 (5%), Eos 0,03 (1%)), platelet count 64 × 10^9^/l, red blood cells 3,66 × 10^9^/l, haemoglobin level 115 g/l (2) liver function tests were raised: AST 2.21 IU/L, ALT 0.95 IU/L and γGT 1.30 IU/L with bilirubin within normal ranges-bilirubin 17 IU/L (3) LDH was raised to 15,68 IU/L; raised was also CRP to 35 (4) serology for HAV showed reactive IgG anti-HAV antibodies, whereas IgM anti-HAV, HbsAg, anti- HBc, anti-Hbs, anti-HCV and CMV were negative.

### Cytologic findings

**Figure 1:**
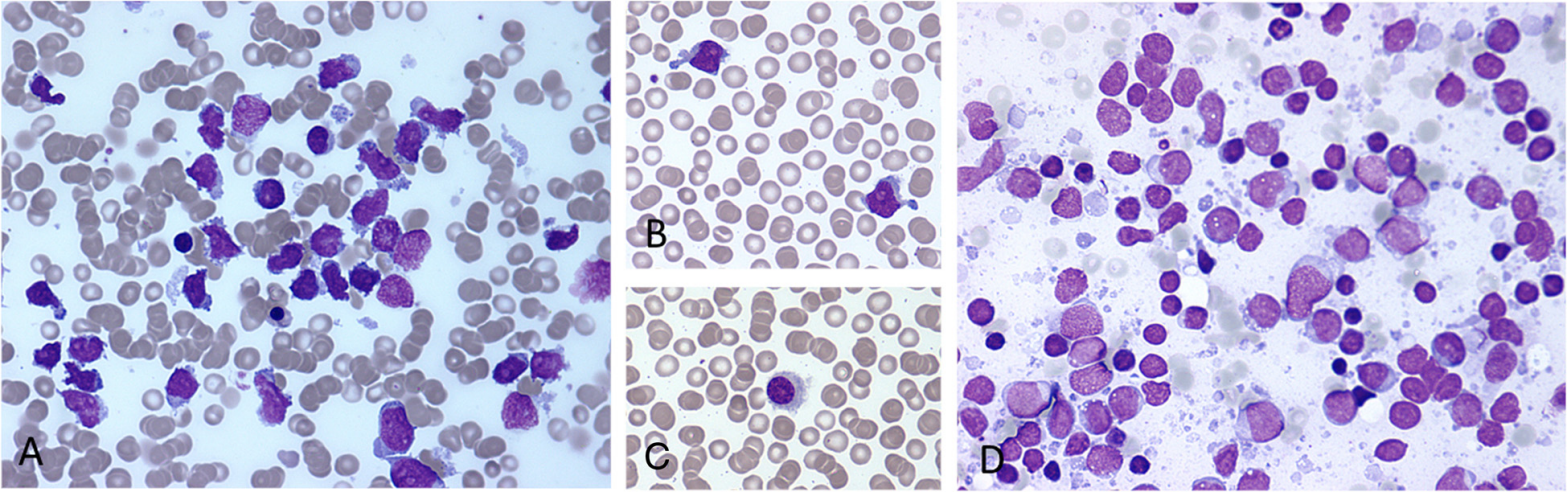
**A-D. Cytological features of composite HSTCL and HCL. A**: HSTCL cells in bone marrow aspirate, MGG, 60×. **B**: HSTCL cells in peripheral blood smear, MGG, 60×. **C**: HCL cell in peripheral blood, MGG, 60×. **D**: HSTCL cells in FNAB lymph node sample, Giemsa, 60×.

**Figure 2:**
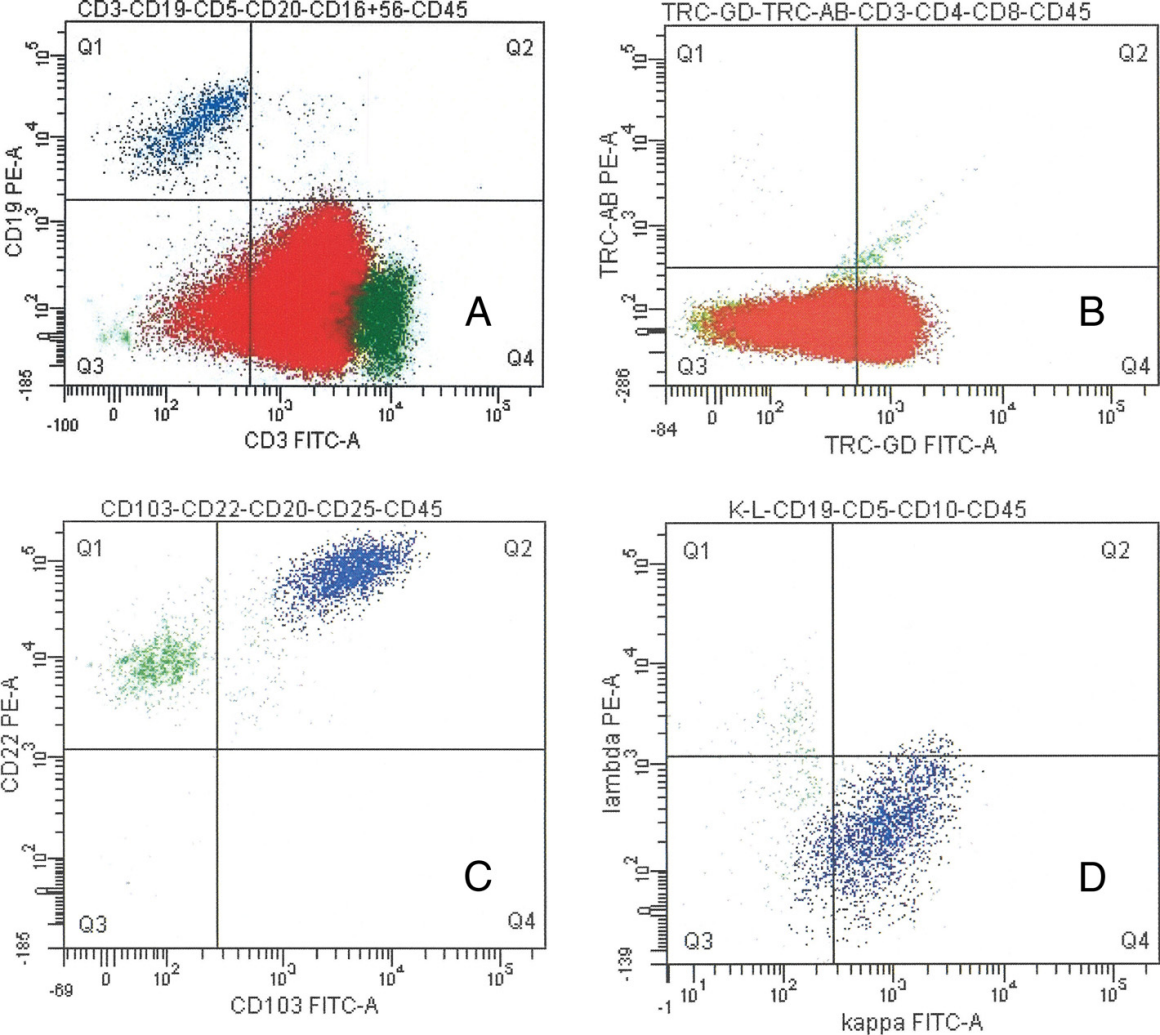
**(Red rectangle symbol) *…HSTCL, *(Blue rectangle symbol) *…HCL, *(Dark green rectangle symbol) *…reactive T-cells, *(Light green rectangle symbol) *…reactive B-cells. ***FCI findings of composite HSTCL and HCL in bone marrow aspirate. **A**: CD3 dim positive population of HSTCL. **B**: γδ T-cell receptor dim positive population of HSTCL. **C**: CD103 and CD22 positive population of HCL. **D**: Monoclonal, kappa positive population of HCL.

The Giemsa-stained smears from bone marrow and peripheral blood showed presence of medium-sized atypical lymphoid cells. These cells slightly differed in size and shape, being round, oval or droplet-like with irregular cell borders. The cytoplasm was scant and basophilic, containing few small vacuoles. The nuclei were round or oval. Chromatin was immature, finely granular, with small inconspicuous nucleoli (Figure 1A, B, D). On flow cytometric immunophenotyping (FCI) of bone marrow and peripheral blood, these cells had immunophenotypic profile consistent with gamma-delta T-cell lymphoma (CD3 dim +, CD7 dim+, CD2+, TCR*γδ* dim +, CD52 dim +, CD56+, CD5-, CD4- and CD8-) (Figures 2A, B). Besides T-cell lymphoma, a small population of monoclonal B-cells with immunophenotype characteristic of HCL (CD103+, CD25+, CD22+, surface-kappa+) (Figures 2C, D) was also present in the bone marrow and peripheral blood. After careful re-examination of peripheral blood smears, only few hairy cells were found. These cells were medium-sized and oval in shape, their cytoplasm was abundant, pale basophilic, with circumferential “hairy” projections (Figure 1C).

### Pathologic findings

**Figure 3:**
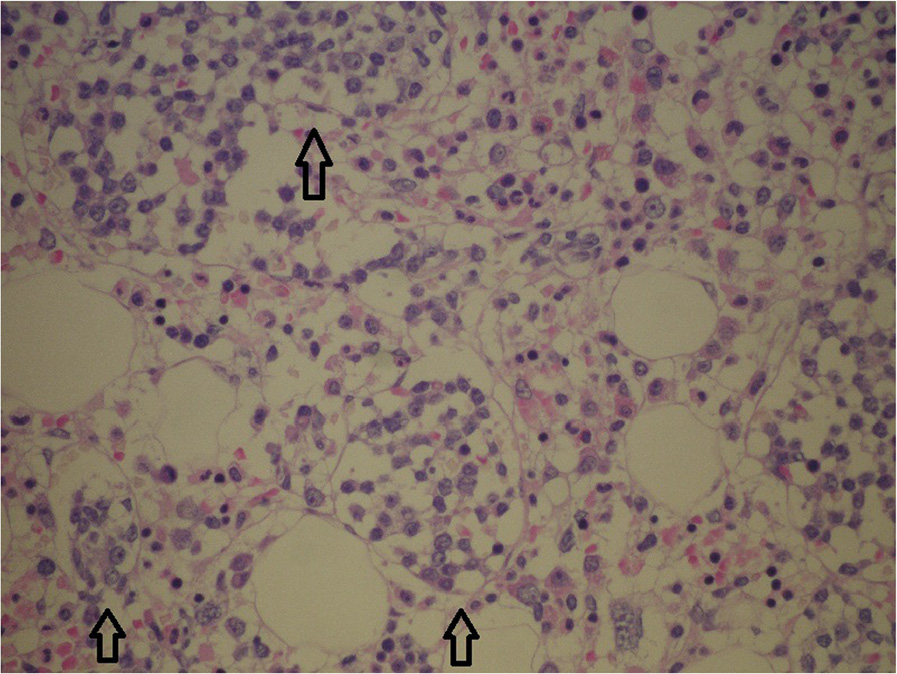
Bone marrow biopsy: H&E, 20×. Interstitial infitrates of HCL cells and distended sinusoides containing HSTCL cells (arrows).

**Figure 4:**
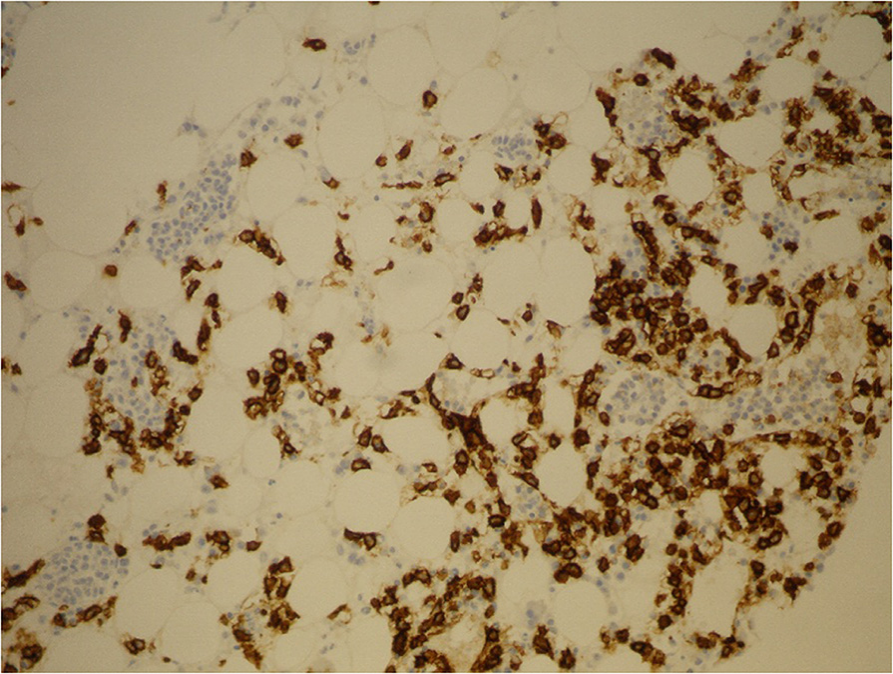
Bone marrow biopsy: CD20 IHC, 20×.

**Figure 5:**
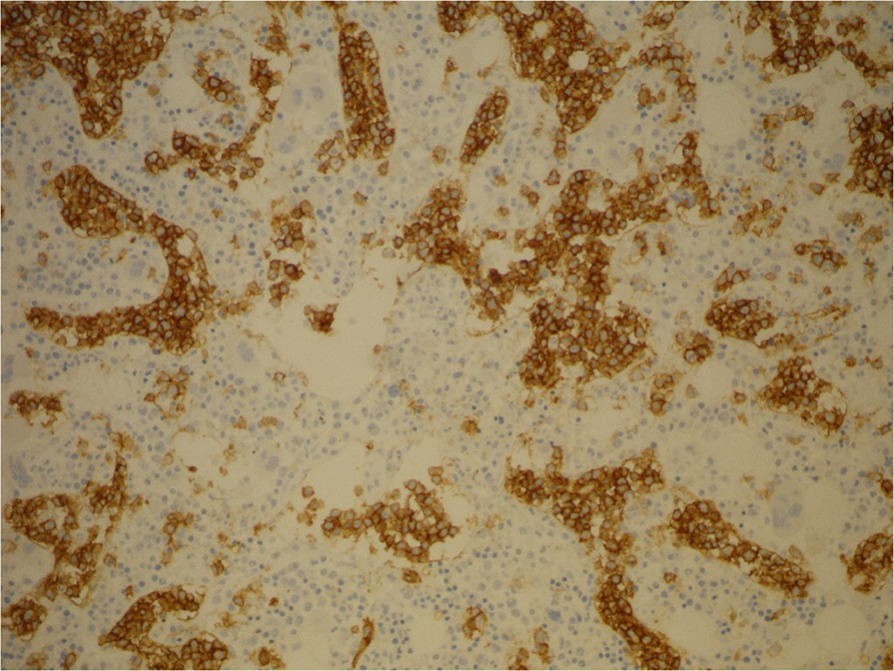
Bone marrow biopsy: CD3 IHC, 20×.

**Figure 6:**
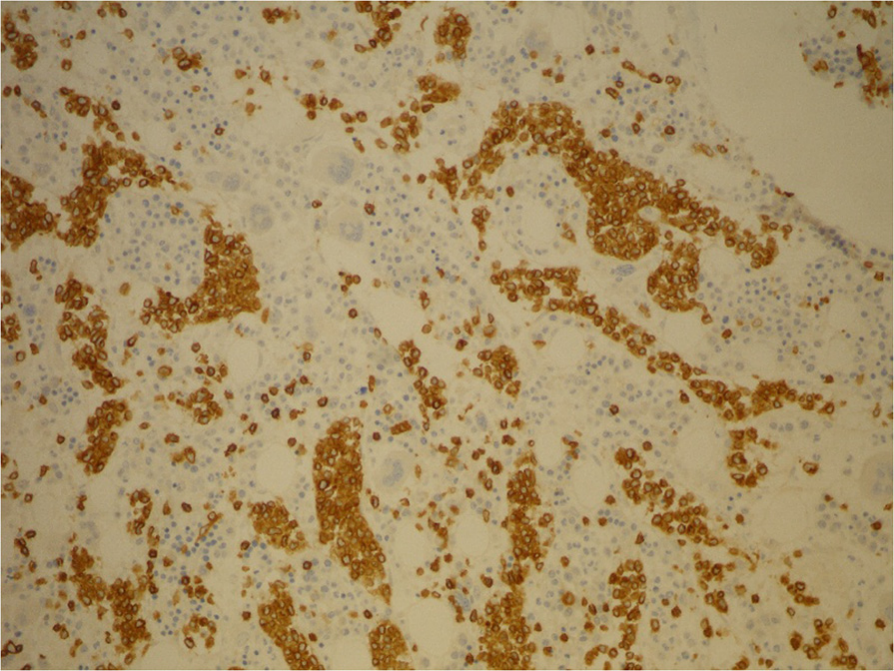
Bone marrow biopsy: CD56 IHC, 20×.

Biopsy of a slightly enlarged left inguinal lymph node and bone marrow trephine biopsy were also performed. Histology of the lymph node showed partial, predominantly intrasinusoidal infiltration by medium-sized, atypical lymphoid cells that were immunohistochemically positive for CD3, CD56, and TIA, while other T- and B-cell markers were negative. Bone marrow biopsy (Figures 3,4,5 and 6) revealed hypercellular bone marrow with reduced orthotopic haematopoiesis and intrasinusoidal infiltrates consisting of medium-sized lymphocytes with the same immunohistochemical profile as was observed on lymph node infiltrates. To continue, patchy interstitial infiltrates were also present, consisting of medium-sized lymphocytes with a round nuclear profile and fine chromatin, and they were widely separated from each other by abundant clear cytoplasm. These cells were positive for CD20, HCL, Cyclin D1 and TRAP (Figures 3 and 4). There was also a marked increase in reticulin fibres. According to the morphology, the immunophenotype and the infiltration pattern of lymphoid infiltrates, the diagnosis of coexistent HSTCL and HCL was established.

### Follow up

**Figure 7:**
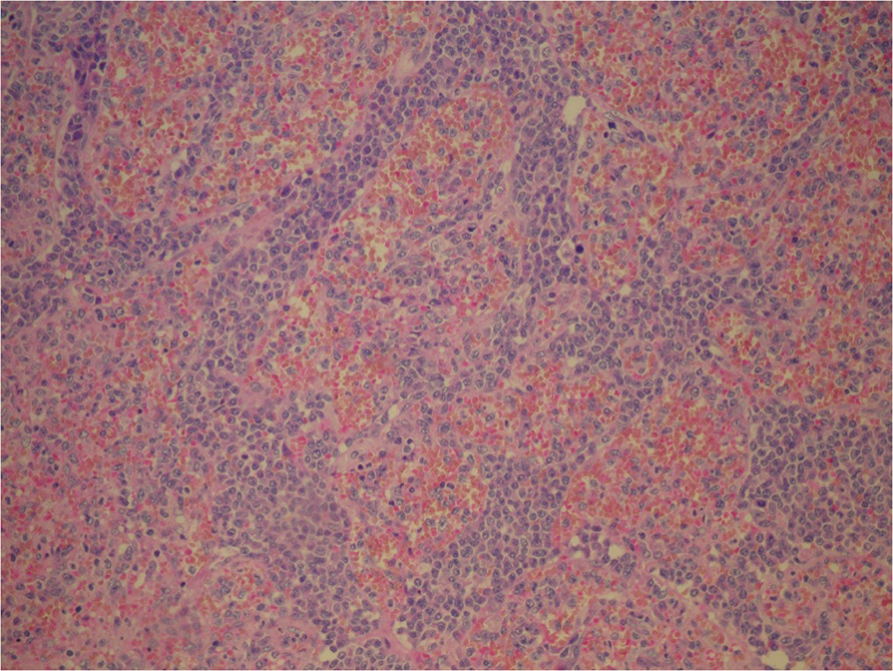
Spleen: H&E, 20×.

**Figure 8:**
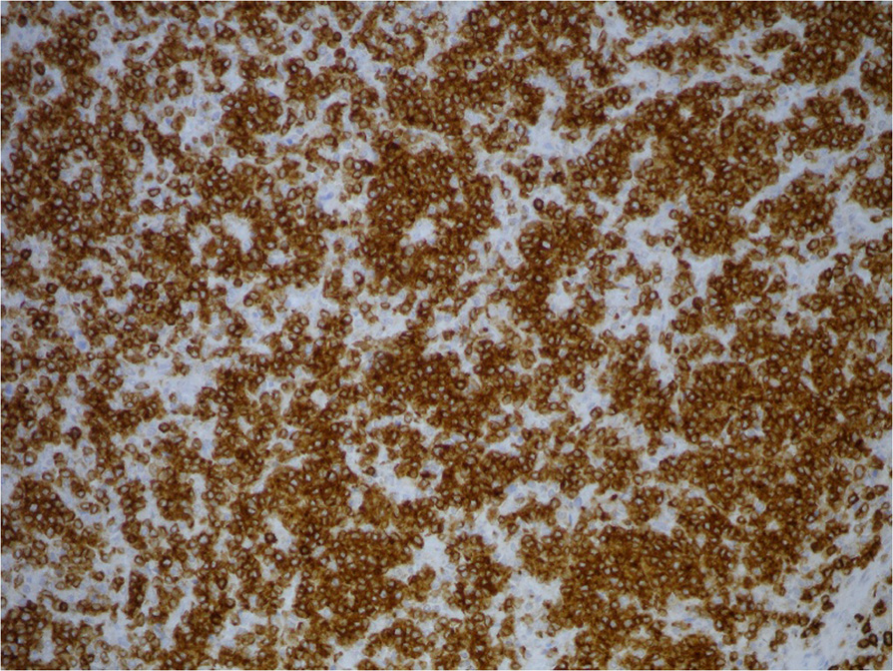
Spleen: CD3 IHC, 20×.

**Figure 9:**
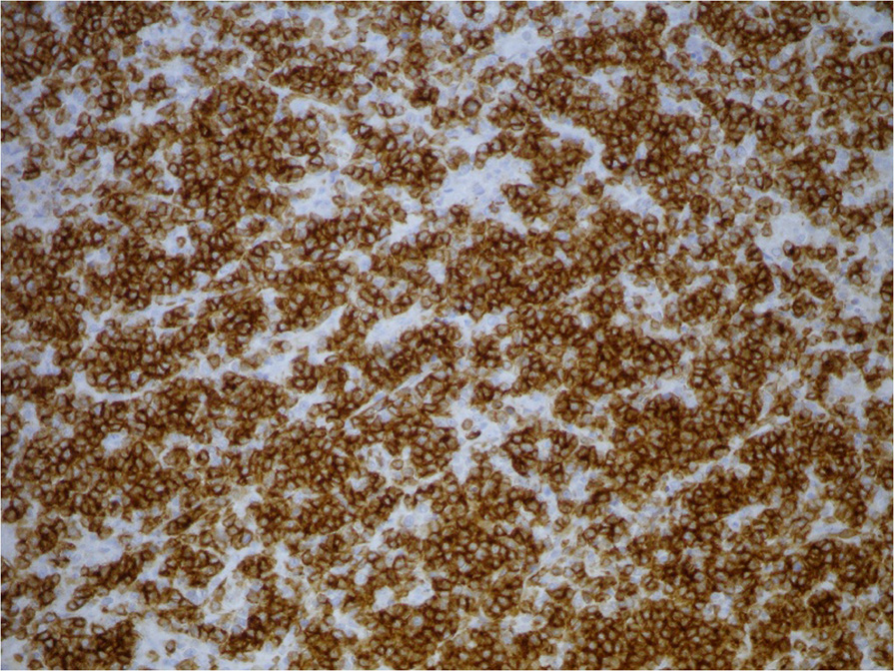
Spleen: CD56 IHC, 20×.

The patient was treated with multivalent chemotherapy including rituximab. The effect of treatment was monitored by clinical and ultrasound examination, leukocyte and lymphocyte counts and FCI of peripheral blood. The patient received the first immunochemotherapy regimen with R-VACPE (rituximab-vincristine, doxorubicin, cyclophosphamide, prednisone, etoposide) and splenectomy (Figures 7,8 and 9). After that HCL regressed completely while HSCTL was refractory to the treatment. R-VACPE was therefore changed to R-DAHP (rituximab, dexamethasone, cytarabine, cisplatin) and then to R-EPOCH (rituximab- etoposide, vincristine, doxorubicin, cyclophosphamide, prednisone). However, all these treatment regiments turned out to be only partially effective. The disease progressed again in the liver, and the patient’s general condition worsened. Therefore, only palliative treatment with liver irradiation was continued. The patient died soon because of haematemesis and Mallory-Weiss syndrome, 7 months after the initial diagnosis.

## Discussion

HCL is an uncommon form of chronic lymphoproliferative disease, characterised by indolent course. Hairy cells typically home to the spleen and circulate only late in the disease. Their proliferation is regulated by different cytokines and growth factors in an autocrine and paracrine fashion [4]. Many manifestations seen in the peripheral blood in patients with HCL are the result of interactions between the neoplastic B cells and the T-cell system. Among others, there are profound alterations in the T-cell immunity with the production of a skewed T-cell repertoire [5], production of autoreactive T cells, and an increase in the CD3 + γδ cells as a persistent clonal excess, as well as monocytopenia, leucopenia, and so on [6]. All of the above-mentioned factors contribute significantly to the immune deficiency found in HCL patients and to the extreme sensitivity to opportunistic infections.

Since all of the above-listed factors play important roles also in the surveillance against neoplasia, it is likely that the appearance of the second neoplasms in HCL reflects an underlying immune deficiency in such patients rather than random coexistence of the two diseases. Some studies showed that patients with HCL have an increased risk of second malignancy, either in a form of synchronous disease [7-10] or in a form of an increased incidence of a second neoplasm after the treatment of HCL [11-13]. Among malignancies with synchronous occurrence, the most frequent is the occurrence with other haematopoietic malignancies [14-21], B-cell lymphomas in the first place, and very rarely also with T-cell neoplasms. Contemporary literature provides only rare case reports describing the coexistence of HCL with LGL leukaemia [20] and simultaneous presentation of HCL and peripheral T-cell lymphoma [19].

On the other hand, HSTCL is a rare and very aggressive form of extranodal lymphoma with a median survival of less than 2 years, despite therapy [1]. Its coexistence with other haematologic malignancies is practically non-existent, which could be a result of its aggressive nature and the usually quick lethal outcome. In the recent literature, there are only two case reports of HSTCL that presented together with some other type of haematologic disease. One is a case of coexistence with aggressive CD56+ CD3- TcRγδ- leukaemia [22], and the other is HSTCL in conjunction with systemic mastocytosis [23].

However, coexistence of two or more morphologically and immunophenotypically distinct lymphoma clones in a single anatomic site is nowadays defined as composite lymphoma [24]. The incidence of composite lymphoma has been reported to vary between 1% and 4.7% [25]. Many different combinations of composite lymphomas have been described [24]. The diagnosis of composite T-cell and B-cell lymphomas is particularly challenging because the neoplastic T-cells may outnumber the neoplastic B-cells, occasionally creating a false impression that B-cells are reactive. Similarly, the neoplastic B-cells may also outnumber the neoplastic T-cells, which may result in overlooking the T-cell lymphoma. A multidisciplinary approach, encompassing immunophenotypic, cytogenetic and molecular analyses, is therefore mandatory to obtain an accurate diagnosis. FCI can be very helpful in these settings, because it can detect small populations of neoplastic B-cells or T-cells, which could be overlooked in morphological and immunohistochemical analyses [26].

Any lymphoproliferative disease infiltrating spleen and liver can mimic HSTCL or HCL. Detailed study of morphology, together with immunophenotyping, gene rearrangement analyses, determining of EBV status and cytogenetic studies could help to establish the right diagnosis [27-32]. Two diseases that show great overlapping with HSTCL regarding clinical picture as well as immunophenotype are aggressive NK-cell leukaemia and advanced extranodal NK/T-cell lymphoma, nasal type. Although immunophenotype could overlap significantly, rearranged TCR gene and no relation to EBV infection are distinguishing features that speak in the favour of the HSTCL [27-29]. HCL can be distinguish from other B-cell neoplasms by its morphology and characteristic immunophenotype, while presence of B symptoms, organomegaly and signs of infection exclude the diagnosis of monoclonal B lymphocytosis with HCL-like phenotype in our patient [33].

In an attempt to explain the coexistence of HCL and HSTCL in our patient, some hypotheses can be formulated. Simultaneous appearance of HCL and HSTCL in our patient could be only fortuitous. However, since HSTCL often arises in the setting of chronic immune suppression, and HCL is a malignancy with a proven profound immune deficiency, their random coexistence is less likely. If persistent clonal excess of T-lymphocytes and skewed T-cell repertoires found in HCL are the only factors that could influence the course of T-cell oncogenesis in HCL patients, one could expect a much higher incidence of synchronous occurrence of HCL and T-cell lymphoma.

## Conclusions

In conclusion, hereby, we present the first case of coexistent HCL and HSTL that has been, to the best of our knowledge, described in the English literature so far.

## Consent

Written informed consent was obtained from the patient’s parent for the publication of this report and any accompanying images.

## Abbreviations

HCL: Hairy cell leukemia; HSTCL: Hepatosplenic T cell lymphoma; FNAB: Fine needle aspiration biopsy; FCI: Flow cytometric immunophenotyping; R-VACPE: Rituximab-vincristine, doxorubicin, cyclophosphamide, prednisone, etoposide; R-DAHP: Rituximab, dexamethasone, cytarabine , cisplatin; R-EPOCH: Rituximab- etoposide, vincristine, doxorubicin, cyclophosphamide, prednisone.

## Competing interests

The authors declare that they have no competing interests.

## Authors’ contribution

**GG and VKP**: Have made substantial contributions to conception and design, acquisition of data, analysis and interpretation of data; have written the manuscript and have been involved in drafting it and revising it critically for important intellectual content. **BG and MV**: Have been involved in drafting the manuscript or revising it critically for important intellectual content. All authors have given final approval of the version to be published.
